# Is Our Newborn Screening Working Well? A Literature Review of Quality Requirements for Newborn Blood Spot Screening (NBS) Infrastructure and Procedures

**DOI:** 10.3390/ijns9030035

**Published:** 2023-06-22

**Authors:** Birgit Odenwald, Inken Brockow, Marianne Hanauer, Anja Lüders, Uta Nennstiel

**Affiliations:** Newborn Screening Centre/State Institute of Health, Bavarian Health and Food Safety Authority, 85764 Oberschleissheim, Germany

**Keywords:** newborn screening, neonatal screening, dried blood spots, screening programme, evaluation, process quality, quality management

## Abstract

Newborn screening using dried blood spots (NBS) is widely acknowledged as a highly successful procedure in secondary prevention. For a number of congenital disorders, severe disability or death are impressively prevented by early detection and early treatment through NBS. However, as with any other screening, NBS can also cause harm, and the principle that “the overall benefits of screening should outweigh the harms” must be considered when introducing and implementing NBS programmes. This publication compiles the results of a systematic literature research on requirements for NBS infrastructure and procedures which was conducted as part of a research project on the quality and shortcomings of the NBS pathway in Germany. The compilation contains the requirements and recommendations for realising the principle of “maximise benefits and minimise harms” in relevant NBS pathway components such as parental education and information, coverage, timeliness, laboratory quality assurance, follow-up of abnormal results, confirmatory diagnostics, documentation, and evaluation. The results reflect the complexity of NBS infrastructure, and thus, they illustrate the importance of considering and implementing NBS as a well-coordinated public health programme with continuous quality management. Special attention should be paid to the perspectives of parents and families. Some NBS issues can substantially benefit from digital instruments or international cooperation. The literature review presented here has contributed to a concept of proposals for the advancement of NBS in Germany, and despite different settings, it may as well be of interest for other countries to achieve the best possible course and outcome of NBS for each child.

## 1. Introduction

Newborn screening using dried blood spots (NBS) is widely reported to be a very successful measure in secondary prevention [1,2,3,4,5,6]. The early detection of treatable congenital diseases via NBS prevents severe disability and death [7,8,9,10], which is a great achievement for those who are affected and their families. Successful NBS requires the diagnosis and initiation of treatment before symptoms appear, i.e., in the first days to weeks of life. This is only possible through screening the entire newborn population in the first days of life, followed by rapid diagnostics. The target diseases of the NBS are rare and affect, for example, in the current German NBS programme, a total of 1 in approximately 800 newborns (less than 0.1% of the newborn population) [11]. This entails that the unaffected “healthy” more than 99.9% of the newborn population must also be screened in order to detect the few that are affected. In many countries, NBS is offered to all newborns as a public health measure. Its most important benefit is the direct health benefit for children diagnosed and treated early as a result of screening. Additionally, in the long term, pre-symptomatic treatment also enables the affected children’s social participation in later years of life and may reduce the burden on their families [12]. Nevertheless, NBS cannot ignore the statement “All screening programmes do harm; some do good as well” [13]. For example, false positive screening results strain the health system and can trigger short- or long-term psychosocial distress for families (anxiety or vulnerable child syndrome), and NBS may cause overtreatment for mild forms of diseases [14,15,16,17,18,19,20]. The ongoing expansion of NBS programmes, which is likely to continue as the use of genetic technologies widens, allows more children to benefit from NBS, but also increases the complexity of NBS structures and the number of potential harms, such as false or unclear findings. Thus, despite the clear benefit of NBS for affected children and their families, the rule of “maximising benefit and minimising harm” [13,21] also stands for NBS programmes in complying with the principle that “the overall benefits of screening should outweigh the harms” [22,23].

In Germany, NBS for 12 congenital metabolic or endocrine disorders was included in the standard benefits of the statutory health insurance funds in 2004, and it has since been expanded to its current 17 target diseases (Table 1). The national Federal Joint Committee (Gemeinsamer Bundesausschuss, G-BA) decides on the introduction of new target diseases on the basis of evidence reviews by the independent Institute for Quality and Efficiency in Health Care (Institut für Qualität und Wirtschaftlichkeit im Gesundheitswesen, IQWiG), as well as reviews of ethical justifiability by the national Gene Diagnostics Commission (§16 GenDG [24]). The German NBS programme is regulated by the national Paediatrics Directive (Kinder-Richtlinie), which was issued by the G-BA [25], and it is subject to the Gene Diagnostics Act (GenDG [24]), which was enacted in 2010. The basic regulations on the German NBS procedure include: written parental consent after the provision of information by a medical doctor, blood sampling between 36 and 72 hours of life (usually in a maternity clinic), analysis by eleven authorised laboratories, and information of parents about abnormal results solely through the sender of the blood sample, i.e., primarily a physician from a maternity clinic [24,25].

**Table 1:** Target disorders for NBS in Germany in 2022 [25].

**Metabolic Disorders:** phenylketonuria (PKU) and hyperphenylalaninemia (HPA), maple syrup urine disease (MSUD), biotinidase deficiency, galactosemia, medium-chain acyl-CoA dehydrogenase deficiency (MCADD), long-chain 3-hydroxyacyl-CoA dehydrogenase deficiency (LCHADD), very-long-chain acyl-CoA dehydrogenase deficiency (VLCADD), carnitine cycle defects, glutaric acidemia type 1 (GA 1), isovaleric acidemia (IVA), and tyrosinemia type 1 **Endocrine Disorders:** congenital hypothyroidism (CH) and congenital adrenal hyperplasia (CAH) **Hemoglobin and Immunodeficiency Disorders:** sickle cell disease (SCD) and severe combined immunodeficiency (SCID) **Neuromuscular Disorder:** spinal muscular atrophy (SMA) **Cystic Fibrosis** (CF)

The German Society for Newborn Screening (Deutsche Gesellschaft für Neugeborenen-Screening, DGNS) publishes annual national quality reports using data from the eleven screening laboratories [26]. These reports, as well as a recently published evaluation of the German NBS 2006–2018 [27], show that NBS is being implemented successfully in Germany, but that there is still room for improvement [28]. 

A research project on the quality and advancement of the NBS infrastructure and procedures in Germany was commissioned by the German National Association of Statutory Health Insurance Funds (Spitzenverband der Gesetzlichen Krankenkassen Deutschlands, GKV-Spitzenverband). While the impact of NBS extensions on infrastructure requirements played a role in the rationale for the tender, the process of evaluation and selection of target diseases was not included in the work commissioned. As one part of this research project, we conducted systematic literature research on the requirements for NBS infrastructure and procedures, and we summarised its main results for this publication.

## 2. Materials and Methods

A systematic literature search on the infrastructure and procedures of newborn blood spot screening (NBS) was completed using the PubMed and Scopus databases for the publication period 2011 to 2021. Particular focus was directed to the issues of programme organisation, quality management, communication, tracking and follow-up, and digital development. The key principles of screening ethics that explain the requirements for the structure and processes were taken into account, but the process of assessing and selecting new target diseases was not systematically researched. As no suitable and uniformly used terms could be identified for a precise search on “newborn blood spot screening”, the search was conducted with a more general search strategy on “neonatal screening” or “newborn screening” in the title or abstract. To reduce the proportion of non-relevant articles on other newborn screening domains, e.g., hearing, hip, or pulse oximetry screening, the results with such terms in their titles were excluded.

The subsequent search strategies that were used for the years 2011 to 2021 included the following:

For PubMed: ((neonatal screening[MeSH Major Topic]) OR (neonatal screening[Title/Abstract]) OR (newborn screening[Title/Abstract])) NOT ((hearing[Title]) OR (heart[Title]) OR (hip[Title]) OR (retinopathy[Title]) OR (biliary[Title])), and for Scopus: (TITLE-ABS-KEY (“neonatal screening”) OR TITLE-ABS-KEY (“newborn screening”)) AND NOT TITLE (hearing) AND NOT TITLE (heart) AND NOT TITLE (hip) AND NOT TITLE (retinopathy) AND NOT TITLE (biliary).

**Figure 1:**
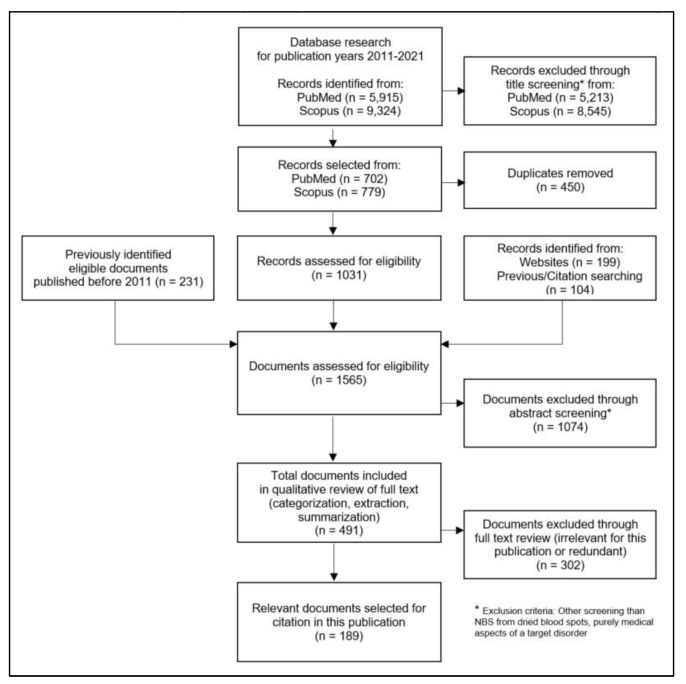
Flow diagram for the systematic literature research on NBS structures and procedures.

Publications where the title and abstract suggested relevant content were imported into a project in the literature management software “Citavi”, and duplicates were removed. We did not consider publications on the purely medical aspects of a single NBS target disease. In addition, websites and internet documents, a collection of NBS literature already held by the authors, which included older publications, and titles that were newly identified from bibliographies were entered into the Citavi project. This was followed by a categorisation using basic NBS aspects and the elements of the NBS process, as well as an assessment of relevance for each document. From the full texts of the documents assessed as relevant, important citations were extracted for the different topics of the analysis, and summaries were prepared. The process of the systematic literature research is shown in Figure 1.

## 3. Results

In the following sections, important results of the systematic literature research on the infrastructure and procedures of NBS from dried blood spots are summarised.

### 3.1. Basic Principles

NBS, as with any screening, is a rough sorting process [21]. A newborn screening test is not intended to be diagnostic; rather, it distinguishes healthy-appearing newborns at higher risk for certain diseases from those at lower risk. In the case of the higher-risk newborns, further examinations are required to either confirm or exclude a diagnosis [21,29,30,31,32,33]. 

Much of the literature on NBS refers to the ten classic screening criteria as outlined by Wilson and Jungner in *Principles and Practice of Screening for Disease*, published by the WHO in 1968 [29] Table 2, and to more recent adaptations, especially that by Andermann [22,23] Table 2. The evolved adapted screening criteria shift the focus from “what to screen” (requirements for suitable target diseases) to “how to screen” (requirements for screening programmes) [34,35]. They include the need for coordination and quality assurance for the screening programmes [34,35], and in particular, they emphasise the medical ethics principle of avoiding harm [36,37,38]. The central ethical guiding principle for screening programmes stated by Andermann—”The overall benefit of screening should outweigh the harm” [22,23]—covers physical, psychological, economic, and social aspects [15,22,23,31,34,35]. This principle must be taken into account in all basic and procedural aspects of a screening programme, as stated and highlighted in the WHO’s 2020 *Short Guide to Screening Programmes*, subtitled “Increase effectiveness, maximize benefits and minimize harm” [21].

As a central guideline with statements and recommendations on NBS based on the screening criteria, the consensus report *Newborn screening in Europe—Expert Opinion Document*, drawn up by an expert board in 2011 upon request by the Council of the European Union on rare diseases, must be particularly emphasised [31,32]. In addition to the recommendations on the selection of target diseases and suggestions for Europe-wide cooperation, this document contains comprehensive guidance on the “best practices” for NBS structures and processes. The *Newborn Screening Follow-up* guideline of the US Clinical and Laboratory Standards Institute (CLSI) [33], last revised in 2013, provides important additions. Therefore, these documents are cited quite frequently in this paper.

### 3.2. NBS Components

#### 3.2.1. Parental Education about NBS and Informed Consent

All relevant documents agree that screening programmes should promote equity and access to screening for the entire target population and ensure informed choice, confidentiality, and respect for autonomy [21,22,31,32]. Thus, screening programmes should provide easy-to-understand information so that people can make an informed decision about participation [21].

Surveys in different countries have found that parents care more about the information on NBS than about their decision-making or consent options [39,40,41,42]. In contradiction to the screening criteria [21,22,34], some authors have advocated that screening for diagnoses with a clear direct benefit for the child (such as PKU, hypothyroidism, MCAD deficiency) can be mandatory or carried out without consent [43,44,45,46,47]. However, regardless of whether screening is mandatory (as in many US states [8,48]) or requires verbal or written parental consent (as in most European states [49] and in line with the screening criteria [21,22,34]), parental information is considered an essential part of NBS [46,50,51,52]. Parents should be given sufficient information to understand the objectives and procedures of NBS [53,54,55] without being overloaded, distressed, or worried [56,57,58]. Parents’ concerns about their baby’s pain from blood sampling, which may even lead to the refusal of NBS [59,60], can be lessened by indicating ways to reduce pain during the heel prick, such as sucking, breastfeeding, oral sweet solution (sucrose or glucose), and body contact/skin-to-skin-care [61,62,63,64,65,66,67,68,69]. Tools such as infographics, videos, and decision aids, as well as risk communication training for healthcare staff, can help to promote understanding and encourage informed decision making [21]. 

Specific recommendations for educating parents about NBS are compiled in Table 3. Several studies in differently organised screening programmes have found that the reality of parent information often does not match the ideal [50,59,70].

#### 3.2.2. Structure and Quality Assurance in the Screening Laboratory

The success of NBS programmes is substantially based on the quality of the screening laboratories and their procedures [83]. There is consensus that screening laboratories should be qualified and certified and that both internal and external quality assurance is required [1,32,84,85,86], and that it should be coordinated at the programme management level [31,32]. Ongoing regular training ensures the competence of the staff and the highest possible analytical quality [21,84,85,87].

In addition to valid test procedures, the valid assessment and reporting of the test results is crucial to the outcome quality because the values of most NBS tests overlap between healthy and affected newborns. Differentiated protocols are recommended for the reporting of test results as highly conspicuous (urgent positive and high risk), borderline (nonurgent positive), or inconspicuous (negative), along with the resulting consequences [5,21,88]. NBS aims not to overlook any affected newborn (the avoidance of false-negative results, high sensitivity) [5,79,89,90]. Simultaneously, because of the burden associated with false-positive results, the proportion of newborns for whom diagnostics can exclude a disease after a positive NBS result should be as low as possible (few false-positive results, high specificity) [17,20,89,90,91,92]. The optimisation of NBS sensitivity and specificity can be achieved by using multiple markers, biochemical or molecular genetic multistage testing procedures (mostly first- and second-tier) [5,35,93,94,95,96,97], and recurring reviews of cut-off values with adjustments, if necessary [83,98]. However, because of the rarity of the diseases screened, data for a sufficient number of confirmed cases to achieve optimal adjustments of all cut-off values are not always available to each laboratory or even to entire countries. Therefore, in recent years, post-analytical multivariate digital interpretation tools that combine data from numerous laboratories, such as the US-based “Collaborative Laboratory Integrated Reports” (CLIR) [98,99,100,101,102,103,104,105,106,107,108] or similar procedures [92,109,110,111], have been increasingly used worldwide with the aim of achieving “precision NBS” with “near-zero false positive rates” [107]. These tools are based on big-data analyses with machine-learning modelling from the digital reports of test results, covariates (such as gestational age, birth weight, and age at blood collection), and final diagnoses. With the inclusion of covariates, the differentiated risk assessment of NBS test results is enabled, and thus, significant improvements in predictive values and reductions in false positive rates can be achieved. Their implementation requires the availability of appropriate digital systems and regulations to ensure data protection [49]. Because of their high potential to improve NBS performance, the use of postanalytical multivariate digital interpretation tools is strongly recommended [49,106,108]. 

#### 3.2.3. Informing Parents about Abnormal (Positive) NBS Results

An abnormal (positive) NBS result means that there is an increased risk for disease and the need for further testing to either confirm or rule out the suspected diagnosis [21,29,30,33], which is distressing and upsetting for the parents of a newborn [79,80,112,113,114,115]. 

Despite all efforts to achieve the highest possible specificity, abnormal NBS results are typically more frequent than confirmed diagnoses [36,79]. Informing the parents appropriately in the case of an abnormal NBS result is a considerable challenge because, in order to achieve the best possible outcome for both the child and the familiy, this involves both emphasizing the urgency for further examinations and avoiding anxiety and distress, as far as is possible [32,36,57,112,116]. Although study results about the extent of the long-term burden on the parent–child relationship caused by false-positive NBS findings are controversial [20,58,114,117,118,119], the content, the ways of communication, and the emotional support of the parents are generally considered important [120,121,122,123]. In order to shorten the stressful phase of uncertainty and to treat affected children early, the time span between notification of the results and the appointment for diagnostics should be as short as possible [32,115,124,125,126]. When reporting an abnormal result to parents, the importance of a skillful and competent person who is well-informed about both the processes and the suspected disease is widely agreed upon [32,116,127,128,129]. It is considered very favourable if the parents are informed by a disease specialist who will then also perform the diagnostics [115,117,124]. Studies have shown that parents prefer to be told by a known person (e.g., their paediatrician or family doctor) [55,130]. However, this is associated with a risk for time delays and is only considered advisable if the informing person is adequately trained, informed, and instructed [32,116,121,131,132]. The European Expert Panel recommends that information content and communication guidelines for the communication of abnormal findings to parents be defined and published centrally at the programme management level. In addition, there is a recommendation that parents also receive information about normal (negative) NBS results, which can enhance quality control and parental well-being [31,32]. Table 4 compiles specific recommendations for informing parents about positive NBS results.

#### 3.2.4. Confirmatory Diagnostics

After a positive NBS result, prompt and guideline-based diagnostics, such as specific differentiated laboratory tests of blood, urine or sweat samples or genetic analyses, must be proficiently performed and interpreted. A confirmed diagnosis requires expert assessment of the disease severity and, if necessary, an early start of therapy. Also, the competent support for the parents from the very beginning, is particularly important. Since all the target diseases of NBS are (very) rare diseases [12] requiring highly competent medical care, all this is best ensured in specialised centres of expertise [88,139,140,141]. Ideally, these centres of expertise should be selected and supervised by medical specialty societies in accordance with objectively defined quality criteria [140,142]. At the programme level, clear case definitions and guidelines for confirmation diagnostics are required for all target diseases [1,31,32,33,81,94,143,144,145,146].

#### 3.2.5. Completeness of NBS Coverage

Ensuring that the offer to perform NBS covers all newborns and that the uptake of NBS is as close to 100% as possible is crucial for the effectiveness of an NBS programme [31,32,33,36,147,148]. In order to identify missed babies and lost test cards, the continuous methodical recording and matching of all births, all received screening cards, and, additionally, parental refusals of NBS must be implemented. This monitoring and cross-checking between born and screened children is best performed digitally, i.e., via databases or information systems [21]. The programme should then include targeted action plans to ensure that unscreened babies for whom there is no documented parental refusal are offered NBS in a timely manner [32,33].

#### 3.2.6. Follow-Up (Tracking) of Positive Results and Requested Repeat Screening Tests

After any abnormal NBS result, a repeat test or confirmatory diagnostics must be performed to confirm or exclude the suspected diagnosis. In some cases, the NBS test may need to be repeated for other reasons, e.g., if the blood sample showed quality deficiencies or was taken too early [33]. As outlined in the CLSI guideline *Newborn Screening Follow-up* [33], to maximise the potential health benefits of NBS, the key tasks for NBS programmes include ensuring that all newborns who require further testing are checked promptly. All actions required to achieve this are termed “(Short-Term) Follow-up” in the CLSI guideline [33] and in several US publications [148,149,150] or “tracking” in other publications [36,149,151,152]. Without systematic tracking, there is a risk that some of the required confirmatory testing will not be completed (“loss to follow-up”), and the affected children will not be treated in time [4,21,33,148,153,154,155]. Information systems with data exchange between medical facilities and laboratories or screening centres can support and accelerate tracking [32]. The structure and extent of tracking programmes must be adapted to the regional organization of NBS and the entire health care system. A centralised tracking model is largely presented as preferable because it presents the best efficiency, controllability, and quality assurance [33,152]. For example, the literature reports loss to follow-up rates of up to 57% without tracking versus 1-2% with a centralised systematic tracking structure [27,153].

#### 3.2.7. Documentation and Evaluation of NBS

The documentation and evaluation of screening programmes, including long-term follow-up for detected patients, are considered essential for continuous quality development in screening [10,33,35,156,157,158,159]. When implementing new screening or expanding existing programmes, outcome studies should be included from the outset [22,35,81,160,161].

Standardised algorithms for confirmatory diagnostics, as well as case definitions for all target diseases, are crucial for the consistent reporting of NBS programmes, monitoring outcomes in newborns with confirmed diagnoses, and evaluating the NBS benefit [32,33,81,94,143,144,145]. Good epidemiological practices include defining all variables of interest precisely and operationalising them in a standardised way [162]. Databases are being requested for monitoring and evaluating NBS programmes [31,32].

Feedback reports from medical institutions to NBS laboratories about the results of confirmatory diagnostics are necessary for the evaluation of key NBS aspects, such as the prevalence of the diseases or the quality criteria of the screening tests [35,156], as well as for laboratory quality assurance and the optimisation of analytics [27,157]. Therefore, systems should be in place to ensure that feedback on confirmed diagnoses and long-term outcomes is available for programme evaluation [31,32].

Since the sensitivity of a screening programme can only be assessed by recording later-diagnosed cases with normal screening results (false-negative screening), NBS programmes should actively seek to identify such cases [33], e.g., via patient registries [27,33,156]. 

Only the systematic long-term follow-up for children detected through NBS enables the evaluation of the true NBS benefits for those affected, their families, and society [10,33,35,81,122,158,163,164]. Long-term follow-up is particularly important for mild disease variants with unclear treatment benefits [18,88,165,166]. Registry-based long-term follow-up is often called for, but it has so far been implemented only rarely [35,158,167,168], although its importance for the continuous optimisation of NBS programmes and for the harmonisation of evidence-based recommendations is undisputed [166,167,168,169,170,171,172]. For rare diseases such as those detected through NBS, patient registries are generally important [142,173] and are best set up in multi-regional or international networks [31,49,140,167,173].

### 3.3. NBS Programme Governance and Quality Management

Given the requirements for the preanalytical, analytical, and postanalytical components of NBS and the uniqueness of the neonatal period, particularly high demands are often placed on NBS process quality [21,22,23]. Even if all target disorders meet the screening criteria, the establishment of a screening programme with optimised and standardised procedures is assessed as necessary to “maximise benefit” and “minimise potential harm”. The statement “newborn screening is a programme and not just a test” is often found in the literature [5,7,21,31,43,45,48,56,81,174]. Andermann calls for “No screening without a screening programme” [175], noting that screening programmes require coordination at the levels of education, testing (such as analytical and clinical validity and laboratory quality assurance), clinical services (such as recruitment, consent, and diagnostics), and programme management (such as supervision, resource management, organisation of services, and monitoring of results) [175,176]. 

Well-informed and trained NBS personnel, both in NBS procedures and in communication, are essential for successful NBS. The correct and timely collection and shipment of blood samples, as well as the careful and complete documentation and transmission of all relevant data, are prerequisites for the conclusive and timely results of NBS laboratory analyses. All healthcare professionals involved in the NBS process therefore require adequate training in accordance with defined contents [21,31,32]. 

In NBS programmes, special attention must be paid to short process times [7,177,178,179] because for some target diseases, there is a risk of life-threatening crises in the first days of life without timely therapy, and for others, the success of a treatment depends on its early start. Therefore, time delays must be prevented for all procedural elements, such as blood collection, shipping, laboratory turnaround, notification of results, and diagnostics, in order to ensure that the affected children can start treatment in time and thus achieve the maximum benefit from NBS [177,179]. 

NBS, as a public health task (e.g., [33,56,180,181]) implies that the entire NBS programme and the entire screening pathway— from education, registration, blood collection, and analysis to treatment and follow-up—should be planned and implemented in accordance with defined criteria in a well-structured and organised programme with continuous reviews and adjustments [2,5,7,21,31,36,81,174,180,182,183]. Implementation should be based on cyclical approaches, with the aim of continual improvement, such as the “Plan-Do-Check-Act (PDCA)” cycle from general quality management [21,184] or the “Public Health Action Cycle” [185], which has been developed in German-speaking areas. The core elements of such ongoing cycles are: situation analysis (assessment), development of strategies to address identified problems (policy development), implementation of the developed strategies (assurance), and review of the effectiveness of the introduced measures (evaluation). These requirements can be found in the European NBS consensus document [31,32], as well as in the WHO guide for screening programmes [21]. 

The European NBS consensus document [31,32] specifically recommends providing defined screening protocols and reviewing these every 1–5 years or as needed, and to monitor the quality of the programme process regularly (possibly annually) so that elements in need of improvement can be identified and appropriate corrective action can be taken. The need for coordination and quality management is explicitly mentioned for the provision of information, training of all professionals involved in NBS, uptake, turnaround times, laboratory procedures, case definitions and diagnostic protocols, databases for monitoring and evaluation, and long-term follow-up.

**Figure 2:**
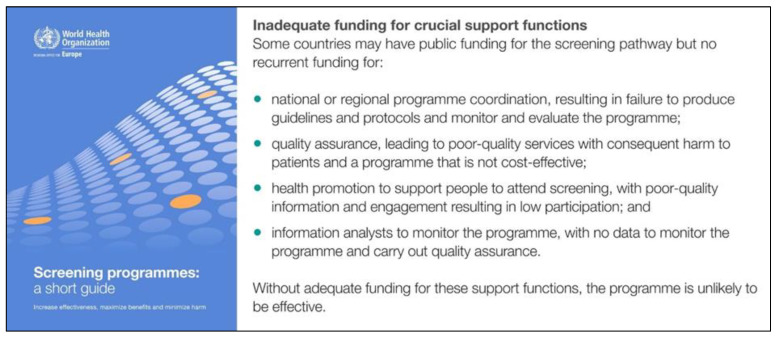
WHO screening guide 2020 [21]: title page and call for funding of ‘crucial support functions’ (p. 37).

The WHO screening guide demands standards, protocols, and guidance based on the best available evidence for each step in the screening pathway, pointing out that the “crucial support functions” of programme coordination, evaluation, and quality assurance systems for all components of screening programmes are often not given enough consideration in the implementation and funding of screening programmes [21] Figure 2.

## 4. Discussion

During a research project on the advancement of NBS in Germany, a systematic literature search on the standards of an up-to-date NBS programme’s infrastructure and procedures was conducted. The review of the current literature indicated that the established fundamental publications on screening and NBS, such as the screening principles by Wilson and Jungner [29] and by Andermann [22], and the European NBS Expert Document [31,32], have not lost their importance and relevance. The fascinating issues of potential new target diseases and the promise and challenges of molecular genetic screening, much covered and debated in the literature, were not a subject of this work. Our results illustrate that the challenges of NBS infrastructure are complex as NBS goes far beyond a “simple blood test”, and these organisational and ethical challenges can hardly be overcome without systematic coordination and quality management [2,5,7,21,31,36,81,174,180,182,183]. The potential offered by digital systems to facilitate, accelerate, and secure the NBS process were particularly highlighted [2,20,49,92,98,99,100,101,102,103,104,105,106,107,108,109,110,111,164,171,186,187,188]. In our view, the aspects presented—although partly seeming rather formal and less fascinating than new perspectives—are essential for existing NBS programmes and grow in importance with further NBS expansions.

In addition to the literature review presented here, the research project included an analysis of the conditions and outcomes of NBS in Germany, as well as structured interviews with various German NBS actors. This showed that currently, German NBS does not meet all the requirements of a “screening programme” in terms of the expanded screening criteria, even if it may be considered successful in many respects, in part due to the great commitment of the various actors. Examples of success include the high ethical standards through early application of the Gene Diagnostics Act and in evaluating of new target diseases (outside the focus of this review), high participation rates, high quality accredited laboratories, and early diagnosis and treatment for most affected children. Nonetheless, the NBS in Germany has some shortcomings in the fields of process coordination, documentation, evaluation, and quality assurance, and it lacks mechanisms to optimise the programme and minimise the potential risks of screening. Digital data exchange systems have so far been seldom used. The implementation of improvements in these areas is hampered by various structural characteristics of the German healthcare system and strict data protection regulations. As part of the research project, recommendations adjusted to the situation in Germany were developed, with a focus on the central coordination of NBS (in German, publication pending). Organisational and structural improvements of the German NBS are needed to ensure that, in addition to the legal framework and the selection of target diseases, all elements of the screening pathway meet ethical requirements.

In our assessment, ensuring that all necessary further testing actually takes place in a timely manner is extremely important for the success of NBS. We therefore consider systematic short-term follow-up, as outlined in the basic CLSI follow-up guideline from the USA [33], to be a central element of NBS programmes. Surprisingly, apart from the CLSI guideline and the few publications on tracking and tracking centres, only a limited amount of literature was found on this topic. Data on loss-to-follow-up rates were only found for a few countries, and even the comprehensive expert consensus document *Newborn screening in Europe* [31,32] does not mention the issue of systematic short-term follow-up (tracking) of positive results and requested repeat screening tests. Even though an efficient follow-up system may be easier to implement in countries with different structures than those in Germany, such as a more centralised or digitalised health care system, NBS short-term follow-up might be an issue that deserves more attention in other regions, as well [153].

Among the central points of the expert document *Newborn screening in Europe* are demands for European consensus-building and cooperation in the field of NBS [31,32]. In later publications, the lack of implementation is lamented [49,189]. Research on NBS programmes in other countries, which was additionally carried out in the context of the present project, showed very clearly that, due to different framework conditions, the many challenges of the procedures and structures of NBS can only be solved on a system- or country-specific basis, even if concepts from other countries can provide valuable suggestions. In the context of the rarity of the screened diseases, however, a database based on sufficient case numbers can best be achieved with international cooperation for many fundamental NBS content issues, such as pilot projects on new target diseases, post-analytical digital instruments, or patient registries. Since, in some European countries, among other things, data protection concerns have thus far hampered the usage of the CLIR digital interpretation tool, with its data server located in the USA, the field of post-analytical multivariate digital interpretation tools combining data from numerous laboratories could greatly benefit from a European cooperation project. Leading approaches to pan-European patient registries already exist [167,173]. 

The authors are aware that it is likely that in other countries, when reviewing the same literature, different emphases may have been set than those in the context of a German project; therefore, some aspects may have been missed in the synthesis. Despite this limitation, the results of the literature review presented in this paper may also be of interest internationally and may perhaps provide ideas for advancements not only in the German NBS but also elsewhere.

NBS is continuously changing due to new diagnostic and therapeutic developments, and it increasingly involves molecular genetics. This implies a challenging opportunity to continuously evaluate and adjust the quality of “old” and “new” components in order to approach the “perfect NBS” as closely as possible. From our perspective, central coordination with continual improvement is essential and critical to the quality of any NBS programme. NBS must be performed on all newborns shortly after birth, which is an extraordinary and vulnerable period for young families, and all efforts to comply with the ethical principle “maximize benefits, minimize harms” should be given extra high priority. Part of the challenge is to continually communicate the benefits of NBS and the great importance of its process quality to all stakeholders and decision-makers so that NBS programmes can help ensure that every infant receives the best possible start in life.

## Figures and Tables

**Table 2:** Classic and emerging screening criteria.

**Wilson and Jungner’s classic screening criteria (1968)** [29] The condition sought should be an important health problem. There should be an accepted treatment for patients with recognised disease. Facilities for diagnosis and treatment should be available. There should be a recognisable latent or early symptomatic stage. There should be a suitable test or examination. The test should be acceptable to the population. The natural history of the condition, including development from latent to declared disease, should be adequately understood. There should be an agreed policy on whom to treat as patients. The cost of case-finding (including diagnosis and treatment of patients diagnosed) should be economically balanced in relation to possible expenditure on medical care as a whole. Case-finding should be a continuing process and not a “once and for all” project.
**Andermann’s Synthesis of emerging screening criteria (2008)** [22] The screening programme should respond to a recognised need. The objectives of screening should be defined at the outset. There should be a defined target population. There should be scientific evidence of screening programme effectiveness. The programme should integrate education, testing, clinical services and programme management. There should be quality assurance, with mechanisms to minimise potential risks of screening. The programme should ensure informed choice, confidentiality and respect for autonomy. The programme should promote equity and access to screening for the entire target population. Programme evaluation should be planned from the outset. The overall benefits of screening should outweigh the harm.

**Table 3:** Compilation of specific recommendations for parental education about NBS.

**Specific recommendations for parental education about NBS Setting:** Education by qualified professionals trained in content and communication [21,31,32] in accordance with specified standards [21,31,32,50]; Optimal timing: prenatal education, possibly during the last trimester of pregnancy [32,33,40,71,72] (postnatal education is characterised as ineffective and unfavourable because parents’ receptivity is often limited in the first days after birth) [50,71]; Preferably personal talk with trained qualified personnel such as a midwife (repeatedly specified as the most suitable person [50,73]), physician, or other, supported by adequate information and material [74]; Adjusted to the level of education, basic content for all parents, with more in-depth content on demand (basic information in a first talk and in clear materials, and then, depending on the parents’ interest, offer of additional in-depth information, e.g., in a further talk or with more detailed materials [32,39,40,73,75]). **Content** [31,32,39,55,76,77,78,79,80]: Aims and benefits of NBS and the impact of NBS on the well-being of affected children; NBS procedure: Heel prick and mentioning ways to minimise baby’s pain during the heel prick [61,62,63,64,65,66,67,68,69]; Possible NBS results and their significance: normal/negative and abnormal/positive results; probability for abnormal findings and the consequent probability of disease; possibility of false-negative and false-positive findings; Ways for and time frame of communicating the findings to the parents; Access routes for additional information. **Media:** Provision of evidence-based information in an adequate language, with translations in several languages [21,31,32]; Innovative information media such as NBS websites or videos are called for [21,46,54,80,81,82] as only using printed educational material (traditional brochures) is increasingly critically viewed [41].

**Table 4:** Compilation of specific recommendations for informing parents about abnormal (positive) NBS results.

**Specific recommendations for informing parents about abnormal (positive) NBS results** [32,105,116,120,124,125,127,128,131,133,134] Structured and supported by scripts or guidelines [31,32,116,117]: ‘The information contents and communication guidelines, for the communication of the need for additional clinical investigations to parents, should be defined at programme management level and published’ [31,32]; If possible, by a specialist [32,115,117,124,130]; otherwise, at least by personnel well-trained in the content and in communication [32,125,135]; Scheduling of an appointment for diagnostics immediately on the day of notification of the results, with the appointment for the next day, at the latest [31,115,116,124,125,127]; Key contents: ○ implications of an abnormal (positive) NBS result and true- and false-positive NBS results [80,116,120] (for example, ‘A ‘screen positive’ result does NOT mean that a baby has the disease. It means that the baby has a higher chance to have the disease and that more testing is needed to find out for sure.’ [136]); ○ precise information about the further steps to be taken [128]; Offers of printed materials or references to reliable websites in addition to verbal information [125,131,135,137,138].
